# CT-Guided Drainage of Fluid Collections Following Liver Resection: Technical and Clinical Outcome of 143 Patients during a 14-Year Period

**DOI:** 10.3390/diagnostics11050826

**Published:** 2021-05-02

**Authors:** Katharina S. Winter, Veronika Greif, Alexander Crispin, Caroline Burgard, Robert Forbrig, Thomas Liebig, Christoph G. Trumm, Robert Stahl

**Affiliations:** 1 Department of Radiology, University Hospital, LMU Munich, 81377 Munich, Germany; Stella.Winter@med.uni-muenchen.de (K.S.W.); Veronika.Greif@med.uni-muenchen.de (V.G.); 2 IBE—Institute for Medical Information Processing, Biometry and Epidemiology, University Hospital, LMU Munich, 81377 Munich, Germany; Cri@ibe.med.uni-muenchen.de; 3 Department of Nuclear Medicine, University Hospital, LMU Munich, 81377 Munich, Germany; Caroline.Burgard@med.uni-muenchen.de; 4 Institute for Diagnostic and Interventional Neuroradiology, University Hospital, LMU Munich, 81377 Munich, Germany; Robert.Forbrig@med.uni-muenchen.de (R.F.); Thomas.Liebig@med.uni-muenchen.de (T.L.); Christoph.Trumm@med.uni-muenchen.de (C.G.T.)

**Keywords:** technical outcome, clinical outcome, CT-guided drainage, liver resection, fluid collection

## Abstract

Purpose: To retrospectively evaluate the technical and clinical outcome of patients with symptomatic postoperative fluid collections following liver resection treated with CT-guided drainage (CTD). Methods: 143 suitable patients were examined between 2004 and 2017. Technical success was defined as (a) sufficient drainage of the fluid collection and (b) the non-occurrence of peri-interventional complications requiring surgical treatment with minor or prolonged hospitalization. Clinical success was defined as (a) decreasing or normalization of specific blood parameters within 30 days after intervention and (b) no surgical revision in addition to intervention required. C-reactive protein (CRP), leukocytes and Total Serum Bilirubin (TSB) were assessed. Dose length product (DLP) for the intervention parts was determined. Results: Technical success was achieved in 99.5% of 189 performed interventions. Clinical success was reached in 74% for CRP, in 86.7% for Leukocytes and in 62.1% for TSB. The median of successful decrease was 6.0 days for CRP, 3.5 days for Leukocytes and 5.5 days for TSB. In 90.2%, no surgical revision was necessary. Total DLP was significantly lower in the second half of the observation period (median 536.0 mGy*cm between years 2011 and 2017 vs. median 745.5 mGy*cm between years 2004 and 2010). Conclusions: Technical success rate of CTD was very high, and clinical success rate was fair to good. Reduction of the radiation dose reflects developments of CT technology and increased experience of the interventional radiologists.

## 1. Introduction

After liver resections, intraabdominal fluid collections frequently occur [1]. The most frequent pathologies are seroma, lymphocele, hematoma or biloma [2]. If infected, abscess formations can cause substantial morbidity and mortality [3]. Computed tomography (CT) is an appropriate method to evaluate fluid collections and to decide about further treatment [4].

In addition to antibiotic therapy, the most appropriate therapeutic method is percutaneous drainage. It allows to precisely target the site of the fluid collection and thereby prevent further complications, for example, necrosis of liver parenchyma [5,6,7].

Percutaneous drainage under CT guidance is a minimally invasive procedure, which is well tolerated by the patients and should therefore be preferred over open surgical drainage [3,8,9]. The technical success rates of percutaneous drainage, as reported in the scientific literature, are very high [5,10,11]. However, various procedure-associated complications of this intervention, such as hemorrhage and sepsis, may occur [6,12,13,14,15].

There are several studies that evaluated the clinical outcome of percutaneous CT-guided drainage (CTD) of intraabdominal abscesses of varying etiologies, including abscesses that were not associated with operation. Most reports confirmed high rates of clinical success [5,11]. A drawback of these studies was rather small patient collectives.

The aim of our study was to retrospectively evaluate the technical and clinical outcome of patients with symptomatic fluid collections following liver resection and treated with CT-guided drainage comprising a large patient cohort in order to enhance the evidence in the assessment of this procedure.

## 2. Materials and Methods

### 2.1. Study Subjects

**Figure 1:**
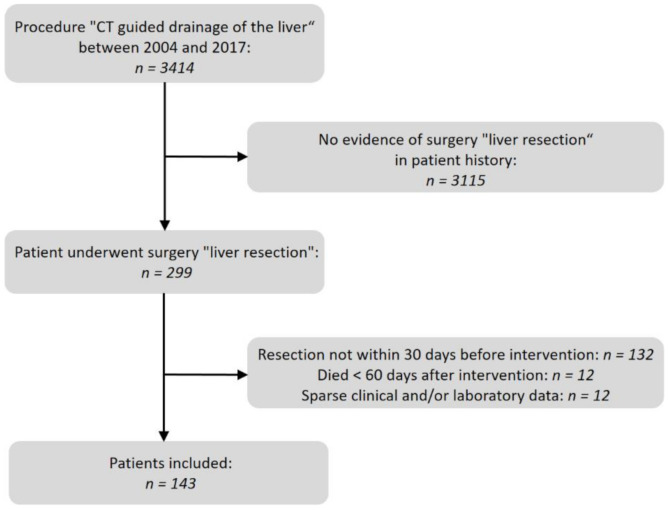
Flow chart of the patient selection process. n: number of patients

A database search of the Radiology Information System (RIS) for the specific procedure key “CT-guided drainage of the liver” in our department was conducted. Indication for previous liver surgery in the results was detected by a full text search of the corresponding written reports as well as in the operations and procedure codes (OPS) of the Hospital Information System (HIS). We evaluated patients with postoperative fluid collections who had undergone liver surgery between 2004 and 2017 and who had received percutaneous CT-guided drainage during a period of max. 60 days after surgery. Details of the selection process are shown in Figure 1.

All interventional procedures performed in this study involving human participants were in accordance with the Helsinki declaration of 1964 and with the ethical standards of the institutional and/or national research committee and its later amendments or comparable ethical standards. Informed consent by the patients or his or her legal guardians to undergo CT-guided drainage was usually obtained 24 hours before the intervention and in case of emergency immediately before the procedure. This retrospective study was approved by the local ethics committee (number 21-0114, Date: 2 February 2021).

### 2.2. CT Imaging Protocol

All interventions were performed on a 64-slice (Siemens SOMATOM 64; Siemens Healthineers, Erlangen, Germany) or on a 128-slice (Siemens SOMATOM Definition AS+/Siemens SOMATOM Definition Edge) CT scanner, respectively. Each patient underwent a pre-interventional CT scan to examine the exact position and size of the fluid collection. The most suitable access path for the percutaneous drainage was planned on these images. Drainage catheters with different diameters (Flexima^®^, Boston Scientific Corporation, Marlborough, MA, USA and ReSolve^®^, Merit Medical, South Jordan, UT, USA; respectively) were used depending on the access path and the experience of the interventionalist. An unenhanced follow-up CT scan was performed immediately after drainage catheter placement to evaluate the outcome of the intervention with respect to the position of the drainage and potential peri-interventional complications. No contrast media was administered. Images were reconstructed using a soft tissue convolution kernel at a slice thickness of 3 mm.

### 2.3. Analysis of Pre- and Peri-Interventional Period

One board-certified radiologist experienced in abdominal imaging with 5 years’ experience assessed surgical techniques (hemihepatectomy, extended hemihepatectomy, minor resection), techniques of intervention (Trocar technique, Seldinger technique), number of drainages, diameter of drainage catheters, access trajectory for drainage (direct, transhepatic, transpleural) and peri-interventional complications (minor, major) according to the Society of Interventional Radiology (SIR) [16]. The mean diameter of the fluid collections was measured. Entities of the fluid collections were not differentiated.

Success in technical outcome after intervention was defined as (a) sufficient drainage of the fluid collection (i.e., leaving less than 10% of the fluid collection after aspiration) and (b) the non-occurrence of peri-interventional complications requiring surgical treatment with minor (<48 h) or prolonged (>48 h) hospitalization [6], respectively.

Inflammatory blood parameters (C-reactive protein (CRP), leukocytes, interleukin-6) and Total Serum Bilirubin (TSB) prior and post-intervention were measured to detect possible superinfections.

The level of the patient radiation dose was provided by the CT scanner for every interventional procedure step by use of the dose-length product (DLP (mGy*cm)). We evaluated the summarized DLP of the pre-interventional planning CT scan, the sum of all intra-interventional CT fluoroscopic acquisitions and of the post-interventional control CT scan, if performed. The resulting DLPs were compared among the time intervals of 2004–2010 and 2011–2017.

### 2.4. Analysis of Post-Interventional Period

Patients receiving reoperation within 60 days after surgery due to insufficient drainage of the fluid collections were evaluated.

The time course of the inflammatory parameters and TSB within 30 days after the intervention was estimated in the subgroup of patients where no evidence of further surgical interventions or complications could be found in the HIS. Based on these results, the success in clinical outcome after the intervention was defined in either a decrease (>50%) of the initially elevated parameters CRP, leukocyte count, and TSB or by the normalization of these elevated parameters within 30 days after intervention, respectively. In addition, clinical success was defined by the absence of necessity for surgical revision related to the intervention. The clinical outcome determined in this way was then compared with the applied surgical techniques to detect possible causal relations.

Microbiological results of the secretion delivered by the drainage catheters were assessed. The number of days until the removal of the drainage was registered for each patient. The removal of the drainage was based on the patient’s clinical and laboratory response [17]. Follow-up imaging was performed only in patients who were not improving clinically [18].

### 2.5. Statistical Analysis

Discrete and continuous data were initially assessed for normality using the Shapiro–Wilk test and by visual inspection of their histograms. Normally distributed variables are provided as mean ± standard deviation (SD). Variables that do not follow normal distribution are shown as median (25%-; 75%-quartiles).

To examine the time course of the blood parameters in the 30-days post-interventional period, the values were log-transformed to achieve normal distribution. Then, generalized linear mixed models (GLMM) were applied. The fixed effect was the number of days after the intervention. Random intercepts were given by subject ID repeated by days. 

The relation between surgical techniques and clinical outcome was assessed with Fisher exact tests. Mann–Whitney tests for independent samples were used to determine significant differences between the radiation exposure, which was found in the two periods. Analysis was performed using R (R Core Team (2020). R: A language and environment for statistical computing. R Foundation for Statistical Computing, Vienna, Austria. URL https://www.R-project.org/, version 4.0.2, accessed on 22 June 2020). A level of significance of alpha = 0.05 was used throughout the study.

## 3. Results

Overall, 143 patients (87 males, mean age 59 ± 14 years) who underwent CT-guided drainage interventions following liver resections between 2004 and 2017 were included in this study. The fluid collections were located at the resection margins. Patients deceased within 60 days after intervention due to reasons unrelated to the drainage procedure and the fluid collection, respectively, were excluded from the analysis. Detailed information on the patient collective is provided in Table 1.

### 3.1. Pre- and Peri-Interventional Analysis

Within an interval of 60 days after surgery, 189 interventions (mean ± SD: 1.3 ± 0.6) per patient were performed. The Trocar technique was used in 174 interventions (92.1%), and the Seldinger technique was used in 15 procedures (7.9%). Mean number of drainages was 1.2 (SD: ±0.4) per intervention. Overall, 222 drainages were inserted. Diameters of drainages were 8 French (F) in 77 cases (39.3%), 10F in 94 cases (48.0%), 12F in 20 cases (10.2%) and 14F in 3 cases (1.7%). For 26 drainages, there was no information on the diameters available. Direct access for drainage catheter placement was used in 131 drainages (67.5%), transhepatic access in 62 drainage placements (32.0%) and transpleural access in 1 intervention (0.5%). For 28 drainages, these data were missing. Detailed information on drainages and techniques of intervention is presented in Table 2.

**Figure 2:**
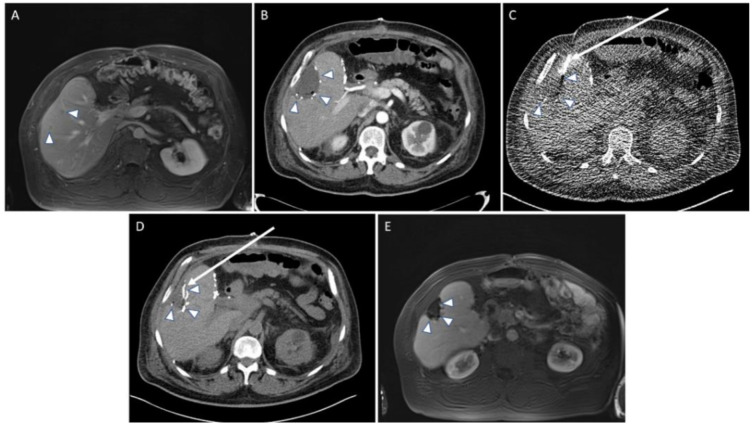
Example of a regular procedure of a CT-guided drainage placement. (**A**) A 64-year-old man with a history of neuroendocrine tumor of the ileum with liver metastasis and previous left side hemihepatectomy. MRI follow-up with hepatobiliary contrast media one year later revealed two new small metastasis in segments 5 and 8 (arrowheads). (**B**) The patient developed fever 5 days after the atypical resection of the liver segments 5 and 8. CT revealed fluid collection and small gas bubbles in the resection cavity, indicating an abscess formation. Arrowheads: resection margins. (**C**) CT fluoroscopic image with drainage (arrow) placement. Arrowheads: resection margins. (**D**) Post-interventional CT control scan. After placement of a 10 F drainage (arrow) and aspiration, the size of the fluid collection becomes significantly smaller. Arrowheads: resection margins. (**E**) MRI follow-up six months later showed unsuspicious resection margins (arrowheads) with granulation tissue.

In 186 interventions, the first placement of the drainage was successful. In two interventions, instant re-placement was necessary. One intervention could not be finished since the fluid collection turned out to be too small for drainage placement. Overall, 188 interventions (99.5%) were technically successful (Figure 2).

**Figure 3:**
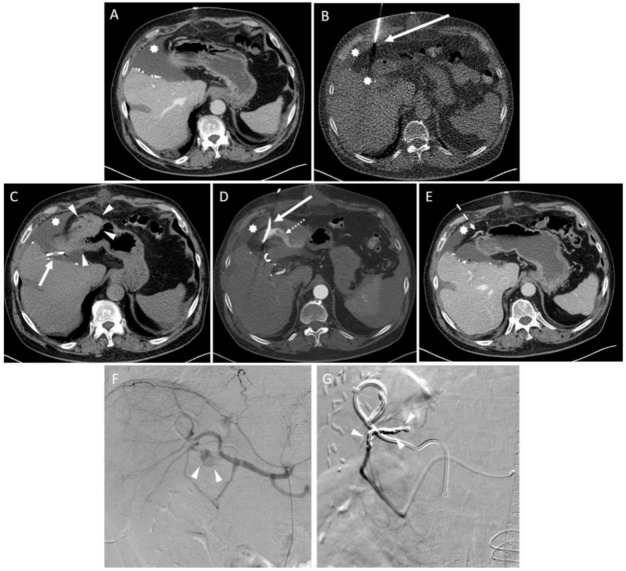
Example of an intervention with complications according to SIR. (**A**) A 72-year-old male after left side hemihepatectomy due to a cholangiocellular carcinoma. Thirteen days after the operation, the patient presented with fever, epigastric pain and elevated inflammatory blood parameters. CT revealed a large fluid collection in the resection area (star). (**B**) CT fluoroscopy-guided placement of a 10 F drainage (arrow) within the fluid collection (star). (**C**) Unenhanced CT post-interventional control scan showed blood collections in the paragastric area and in the right upper quadrant (arrowheads). Arrow: drainage. Star: fluid collection. (**D**) An additional CT scan with arterial contrast revealed extravasation (dotted arrow). Arrow: drainage. Star: fluid collection. (**E**) Workup of the incident showed an injury of the right gastroepiploic artery (dashed arrow). The reason was a restless and uncompliant patient. Due to patient movements, the trajectory was not carried out as planned. Star: fluid collection. (**F**) An immediately performed digital subtraction angiography (DSA) confirmed extravasation of contrast agent from the right gastroepiploic artery (arrowheads). (**G**) Treatment of the bleeding was performed with an endovascular placement of seven microcoils in a sequence (arrowheads).

Peri-interventional complications occurred in seven patients (4.1%), including four major complications according to SIR (Table 3, Figure 3); operative revision was necessary for two patients.

CRP at the day of the intervention (baseline) was 11.4 ± 6.8 mg/dL, leukocytes were 12.6 ± 6.4 × 10^9^/L. nterleukin-6 (median (25%, 75% quartile)) was 143,9101.4, 358.3) pg/dL and TSB was 1.2 (0.7, 2.1) mg/dL.

There were increased baseline levels of CRP (>0.5 mg/dL) in 131 interventions (91.6%), of leukocytes (>9.8 × 10^9^/L) in 60 interventions (51.5%) and of TSB (>1.0 mg/dL) in 58 interventions (44.6%), respectively. Interleukin-6 (>5.9 pg/dL) was elevated in 24 interventions. 

Sepsis was detected in 16 patients (11.2%). However, the development of sepsis cannot solely be correlated to the intervention in any case as most patients were multi-morbid.

DLP (median (25%, 75% quartile)) of the whole intervention was 745.5 (539.8, 1096.3) mGy*cm between 2004–2010 and 536.0 (406.0, 787.5) mGy*cm between 2011–2017, which was significantly different (Mann–Whitney-test *p* < 0.05). Comparing the parts of the intervention, there was a significant (Mann–Whitney-test *p* < 0.05) reduction of radiation dose in pre- (years 2004–2010: 345 (242,617) mGy*cm vs. years 2011–2017: 280.0 (194.5, 406.5) mGy*cm) and intra-interventional (years 2004–2010: 85.5 (43.5, 221.3) mGy*cm vs. years 2011–2017: 28.0 (17.8, 60.3) mGy*cm) CT scans.

**Figure 4:**
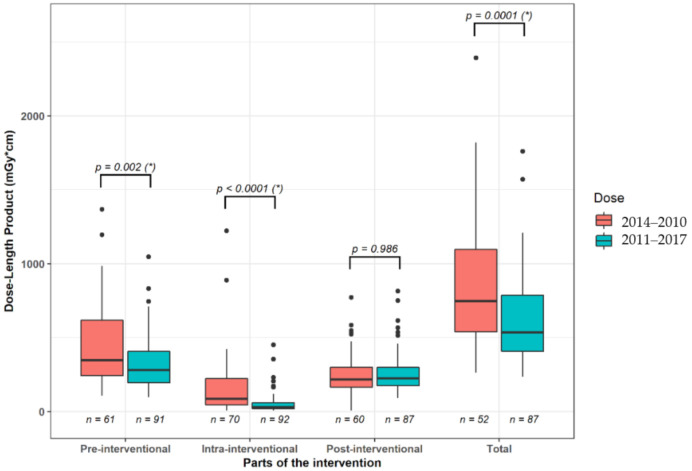
Boxplots of the median radiation dose between the time intervals of the years 2004–2010 and years 2011–2017 for parts of the interventional CT scan and for the whole procedure. (*) indicate significant group differences.

The highest decrease was observed for CT fluoroscopy with a reduction of −67.3% of the median value (Figure 4). DLP for the post-interventional control scan did not differ significantly (years 2004–2010: 217 (164.3, 300.3) mGy*cm vs. years 2011–2017: 223 (174,298) mGy*cm; Mann–Whitney-test *p* > 0.05).

### 3.2. Post-Interventional Analysis

Twenty-five patients had to be excluded from further analysis. Twelve patients deceased within 60 days after intervention for reasons not related to the intervention, for example, respiratory insufficiency. In 12 patients, data of laboratory values were too sparse or inconsistent. In one patient, CT-guided drainage was not successful. Overall, 116 interventions could be included in the post-interventional analysis.

**Figure 5:**
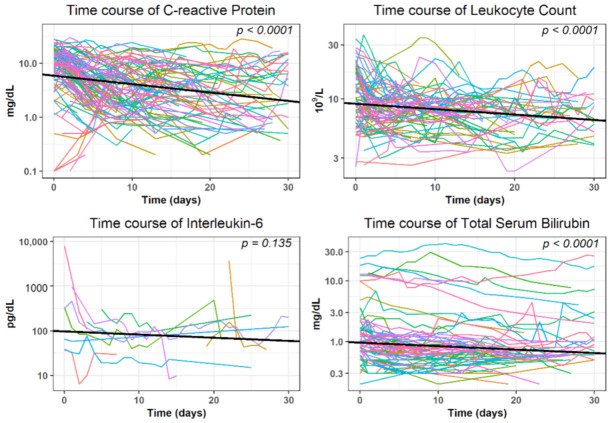
Development of laboratory parameters within 30 days after the intervention in subjects with no evidence of further surgical interventions or complications in the patient record. Please add short explanations titles for (**A**–**D**).

The time course of CRP, leukocytes and TSB revealed a statistically significant (*p* < 0.05) decrease within 30 days after intervention when analyzed with GLMMs in the subgroup of patients with no evidence of further surgical interventions or complications. The log-transformed values decreased with an average of −0.00574 mg/dL for TSB, −0.01559 mg/dL for CRP and −0.00483 × 10^9^/L for leukocytes, respectively (Figure 5 and Table 4). However, values of Interleukin-6 showed no significant decrease in this subgroup; therefore, this factor was not included in our further evaluations. 

Clinical success by our definition of decrease of initially elevated laboratory parameters was reached for CRP in 97/131 interventions (74.0%) after (median (25%, 75% quartile)) 6.0 (4.0, 10.0) days, for leukocytes in 52/60 interventions (86.7%) after 3.5 (2.0, 8.0) days and for TSB in 36/58 interventions (62.1%) after 5.5 (2.8, 11.0) days, respectively. The distribution of the success rate among the different applied surgical procedures in the patients is shown in Table 5. For CRP, the values were relatively close to each other. Patients with right-sided hemihepatectomy had the worst outcome of the success rate for leukocytes and TSB (33.3% and 44.4%, respectively). However, findings were not statistically significant (Fisher exact test *p* > 0.05).

With regard to a successful clinical outcome in terms of a necessary reoperation, in 14 (9.8%) patients, a surgical revision had to be conducted due to insufficient drainage of the fluid collection. This comprised five patients with extended hemihepatectomy and nine patients with minor resections and was not statistically significant (Fisher exact test *p* = 0.089).

Microbiological results of wound secretions were positive in 81 (74.3%) and negative in 35 (32.1%) patients. The most common strains of detected bacteria were Enterococci in 54 patients and Staphylococci in 29 patients, respectively. The most frequent pathogen of fungus infection was Candida in 36 patients. For a detailed presentation of the microbiological results, please see Supplementary Figure S1.

The distribution of the success rate between infected and non-infected fluid collections is shown in Table 6. The proportion of successfully reduced laboratory parameter values tended to be higher in patients with infected fluid collections and was highest with 82.9% for leukocyte count.

Two out of thirty-five (2.9%) patients with non-infected fluid-collections underwent surgical revision due to insufficient drainage. This was a lower rate than in patients with infections, where in 11/81 (13.6%) patients, resurgery was performed. However, these findings were not statistically significant (Fisher exact test *p* > 0.05).

The mean duration time until the removal of the drainage was 14.2 ± 13.7 days.

## 4. Discussion

Hepatic resection has had an impressive growth over time and has been widely performed for the treatment of various liver diseases, such as malignant tumors, benign tumors, and abscesses [19]. The management of complications is challenging. Formation of intraabdominal fluid collections like abscess, biloma, lymphocele, hematoma and seroma following liver resection frequently occur [1]. Its incidences show larger ranges of variation in the literature: for example, Jin et al. reported an occurrence of bile leakage between 4% and 17% [19]. Brustia et al. found postoperative fluid collections after liver surgery in 53.6% of their patients with 12% requiring drainage [20]. In the population studied by Benzoni et al., hepatic abscess was observed in 25% [21]. Abscesses, in particular, are associated with significant morbidity and mortality [4,8], requiring an early and successful treatment. Ultrasound-guided drainage is advantageous if the fluid collection can be confidently visualized. The procedure can then be monitored in real time, without radiation exposure and at low cost. However, it is highly dependent on the experience of the operator and is often not suitable for liver abscesses located in difficult-to-access sites. In addition to antibiotic therapy, CT-guided percutaneous drainage, therefore, has become an alternative for surgery and is currently the standard of treatment [5]. Compared to open surgical drainage, it is less invasive, more cost-effective and can be used repeatedly [9]. Moreover, CT-guided drainage enables both identification of causative microorganisms and targeted treatment in cases of superinfection.

Our retrospective study spanned a period of 14 years and included a large cohort of patients who received CT-guided percutaneous drainage interventions after liver resections. A total of 143 patients with a total of 189 interventions were analyzed in terms of technical and clinical outcomes. Most patients received one drainage per intervention. The trocar technique as a single-step procedure is easier and more economical to apply compared with the drainage procedure conducted using Seldinger techniques and was more frequently used in our patients [22]. As the postoperative fluid collections were mostly superficial, direct access (67.5%) was more frequently used than transhepatic (32.0%) or transpleural access (0.5%), respectively.

CT-guided drainage procedures may result in various complications, such as pneumothorax, hemorrhage, sepsis or death [5,12,13,15]. Prior studies reported complication rates of around 10% [23]. In our study, the rate of peri-interventional complications was slightly lower as they occurred in seven patients (4%), including four major complications according to SIR [16]. In terms of technical success, 98% of interventions were successful in our study. This is in agreement with other authors who reported a high technical success rate of interventional drainage of abdominal abscess with a range of 95–100% [7,10,11].

The cure rates of abdominal abscesses depend on their complexity. Simple abscesses that are unilocular and discrete are reported to be cured in more than 90% of the cases, whereas more complicated collections, such as those with enteric fistulas or pancreatic abscesses, show cure rates of 65% to 90% [5].

In our study, a successful clinical outcome was defined as either a decrease of elevated inflammation parameters (>50%) CRP, leukocyte count and TSB or normalization of these parameters within 30 days after the intervention, respectively. Interleukin-6 was excluded since a preceding analysis in a subgroup of patients with no apparent complications or abnormalities in the patient record showed that this parameter did not decrease significantly during this follow-up period in our cohort. However, this is possible due to the small number of 14 patients (*n* = 14) for which the corresponding interleukin-6 values were available from the records. Clinical success according to the above definition was most frequently observed for the parameter leukocytes (86.7% of the interventions), followed by CRP (74.0%) and TSB (62.1%). Clinical success often occurred within the first week (median: after 3.5 days for Leukocytes, 5.5 for TSB and 6.0 days for CRP, respectively). Furthermore, in our study, it was considered as a clinical success if surgical revision related to the intervention was not necessary (90.2%). Clinical success rates determined with our criteria are comparable to the findings of other authors: Akinci et al. investigated the efficacy of percutaneous drainage of intraperitoneal abscesses in 255 patients. Initial cure rates were 68%, defined as complete healing without any need for recatheterization [23]. In contrast to our study, abscesses were drained either with fluoroscopic, sonographic or CT guidance. In another study, which evaluated 47 patients with 54 drainages in abdominal, retroperitoneal and pelvic abscesses, respectively, clinical success was achieved in 94% of the patients [7]. Lagana et al. evaluated 95 patients with 107 abdominal and pelvic abscesses and obtained clinical success in 92% of the cases [11].

We did not find a significant correlation comparing clinical outcome and preceding operation techniques. This indicates that the postoperative course of the patients was not negatively influenced by more extensive surgical techniques.

In our study, a significant reduction of median DLP was observed in the pre- and peri-interventional CT scans comparing the years 2004–2010 and the years 2011–2017, especially for CT fluoroscopy. This is probably due to ongoing technical developments of CT, including accelerated image acquisition, improved spatial resolution, tube current modulation and the improvement of image quality by iterative image reconstruction [24,25,26]. Another point is the more frequent use of CT-guided drainage over time, leading to increased experience of the interventional radiologists [27]. This reduces the CT acquisition time that is required for trajectory planning and drainage placement.

Regarding microbiological results, the most frequently isolated bacteria of wound secretions of the drainages were Enterococci, followed by Staphylococci, Klebsiella pneumoniae and *E. coli*. The most frequent pathogen of fungus was Candida. Overall, this corresponds to pathogens that commonly predominate in intraabdominal infections [28,29]. In our study, infected fluid collections showed a tendency for higher clinical success rates in comparison to non-infected fluid collections when treated with CT-guided drainage. This could be an indication that drainage is slightly more effective by relieving pus than by removing a solely mechanical accumulation of fluid, such as in a biliary leak.

Several limitations of our study have to be considered. First, we included the occurrence of sepsis within the outcome analysis. Since most patients were multimorbid, the development of sepsis cannot be clearly assigned to the intervention. Second, we did not differentiate between the entities of the fluid collections since this was not the aim of our study. Third, several patients had to be excluded retrospectively from analysis due to missing or incomplete data.

## 5. Conclusions

In conclusion, patients with symptomatic fluid collections following liver resection who underwent percutaneous CT-guided drainage showed a very high technical success rate (99.5%) and a fair-to-good clinical success rate, when assessed by the decrease of blood parameters (CRP: 74.0%, leukocytes 86.7%, TSB 62.1%, respectively) or the absence of necessity for surgical revisions related to the intervention (90.2%). Results were not influenced by the complexity of preceding surgical procedures. Compared to open surgery drainage, CT-guided drainage involves far fewer complications and can be used repeatedly. In addition, it enables both pathogen identification and targeted treatment in cases of superinfection. Technical developments of CT and increased experience of the interventional radiologists resulted in reduced radiation exposure over the years.

## Figures and Tables

**Table 1:** Population characteristics in 143 patients having undergone CT-fluoroscopy guided percutaneous drainage of fluid collections following liver resection.

Variable	
Age (years)	59.8 ± 14.1 (16–83) **^1^**
Sex	
Female	56 (39.2) **^2^**
Male	87 (60.8) **^2^**
Max. Diameter of the fluid collection (cm)	8.7 ± 3.3 (3.0–20.0) **^1^**
**Indications for Liver Resection**	
Malignant disease:	*125 (87.4%)* **^2^**
Liver metastases	70 (49.0%) **^2^**
Hepatocellular carcinoma	30 (21.0%) **^2^**
Klatskin Tumor	11 (7.7%) **^2^**
Cholangiocellular carcinoma	9 (6.3%) **^2^**
Other	5 (3.5%) **^2^**
Benign disease:	*13 (9.1%)* **^2^**
Echinococcus cyst	6 (4.2%) **^2^**
Focal nodular hyperplasia	4 (2.8%) **^2^**
Liver adenoma	2 (1.4%) **^2^**
Hemangioma	1 (0.7%) **^2^**
*Inflammatory*	*5 (3.5%)* **^2^**
**Surgery Techniques**	
Extended hemihepatectomy	36 (25.2%) **^2^**
Hemihepatectomy	44 (30.8%) **^2^**
Minor resection	63 (44.0%) **^2^**

^**1**^: Mean value ± standard deviation (range), ^**2**^: Numbers (Percentage)

**Table 2:** Information on drainages and intervention technique.

Drainages per Intervention (*n*)	Quantity (*n*, %)
1	157 (83.1%)
2	31 (16.4%)
3	1 (0.5%)
Diameter (French)	Drainages (*n*, %)
8	77 (39.3%)
9	1 (0.5%)
10	94 (48.0%)
11	1 (0.5%)
12	20 (10.2%)
14	3 (1.5%)
Technique	Interventions (*n*, %)
Trocar	174 (92.1%)
Seldinger	15 (7.9%)
Access path	Drainages (*n*, %)
Direct	131 (67.5%)
Transhepatic	62 (32.0%)
Transpleural	1 (0.5%)

*n*: number; %: percentage.

**Table 3:** Peri-interventional complications according to SIR.

Type of Complication	Interventions (*n*, %)
**Minor complications:**	3 (1.6%)
Small pneumothorax	3
**Major complications:**	4 (2.1%)
Severe pneumothorax (requiring surgical management)	1
Hemorrhage	1
Laceration (colon, liver)	2

*n*: number; %: percentage.

**Table 4:** Parameters of the generalized linear mixed models used in Figure 4.

	CRP			Leukocyte Count			Interleukin-6			Total Serum Bilirubin		
Predictors	Estimates	CI	*p*	Estimates	CI	*p*	Estimates	CI	*p*	Estimates	CI	*p*
(Intercept)	0.77	0.70–0.84	**<0.001**	0.96	0.92–0.99	**<0.001**	1.99	1.73–2.24	**<0.001**	−0.02	−0.10–0.06	0.656
Time (days)	−0.02	−0.02–−0.01	**<0.001**	−0.00	−0.01–−0.00	**<0.001**	−0.01	−0.02–0.00	0.132	−0.01	−0.01–−0.00	**<0.001**
**Random Effects:**												
σ^2^	0.10			0.02			0.11			0.02		
τ_00_	0.12 _Subject ID_			0.02 _Subject ID_			0.18 _Subject ID_			0.16 _Subject ID_		
ICC	0.54			0.51			0.61			0.88		
N	112 _Subject ID_			88 _Subject ID_			14 _Subject ID_			108 _Subject ID_		
Observations	835			609			112			635		
Marginal R^2^/ Conditional R^2^	0.076/0.572			0.041/0.526			0.013/0.615			0.013/0.884		

CI: Confidence interval; R^2^: Coefficient of Determination; σ^2^: distribution-specific variance; τ00: between-subject variance; ICC: intraclass correlation coefficient, N: number of subjects. P-values in bold indicate significant effects.

**Table 5:** Distribution of the success rate in terms of decreasing laboratory parameters among the different applied surgical procedures.

	C-Reactive Protein			Leukocytes			Total Serum Bilirubin		
Operation Technique	Elevated (*n*)	Success (*n*, %)	No Success (*n*, %)	Elevated (*n*)	Success (*n*, %)	No Success (*n*, %)	Elevated (*n*)	Success (*n*, %)	No Success (*n*, %)
Extended Hemihepatectomy	29	20 (69.0)	9 (31.0)	16	11 (68.8)	5 (31.2)	17	14 (82.4)	3 (17.6)
Left Hemihepatectomy	14	9 (64.3)	5 (35.7)	6	5 (83.3)	1 (16.7)	7	5 (71.4)	2 (28.6)
Right Hemihepatectomy	20	13 (65.0)	7 (35.0)	9	3 (33.3)	6 (66.7)	9	4 (44.4)	5 (55.6)
Minor Resection	43	34 (79.1)	9 (20.9)	21	16 (76.2)	5 (23.8)	15	11 (73.3)	4 (26.7)
Total	106	76 (71.7)	30 (28.3)	52	35 (67.3)	17 (32.7)	48	34 (70.8)	14 (29.2)

*n*: number; %: percentage.

**Table 6:** Distribution of the success rate in terms of decreasing laboratory parameters between infected and non-infected fluid collections.

	C-Reactive Protein			Leukocytes			Total Serum Bilirubin		
Fluid Collection Infection Status	Elevated (*n*)	Success (*n*, %)	No Success (*n*, %)	Elevated (*n*)	Success (*n*, %)	No Success (*n*, %)	Elevated (*n*)	Success (*n*, %)	No Success (*n*, %)
Infected	79	61 (77.2)	18 (22.8)	41	34 (82.9)	7 (17.1)	33	22 (66.7)	11 (33.3)
Non-infected	31	23 (74.2)	8 (25.8)	11	6 (54.5)	5 (45.5)	15	9 (60.0)	6 (40.0)
Total	110	84 (76.4)	26 (23.6)	52	40 (76.9)	12 (23.1)	48	31 (64.6)	17 (35.4)

*n*: number; %: percentage.

## Data Availability

The data presented in this study are available upon reasonable request from the corresponding author.

## Supplementary Materials

The following are available online at https://www.mdpi.com/article/10.3390/diagnostics11050826/s1, Figure S1: Microbiological results in wound secretions divided into the different strains of bacteria and fungi.
