# Supplementary figures and images for: CT-Guided Drainage of Fluid Collections Following Liver Resection: Technical and Clinical Outcome of 143 Patients during a 14-Year Period
Source: Diagnostics (Basel). 2021 May 2;11(5):826. doi: 10.3390/diagnostics11050826 (PMC8147601; doi:10.3390/diagnostics11050826)

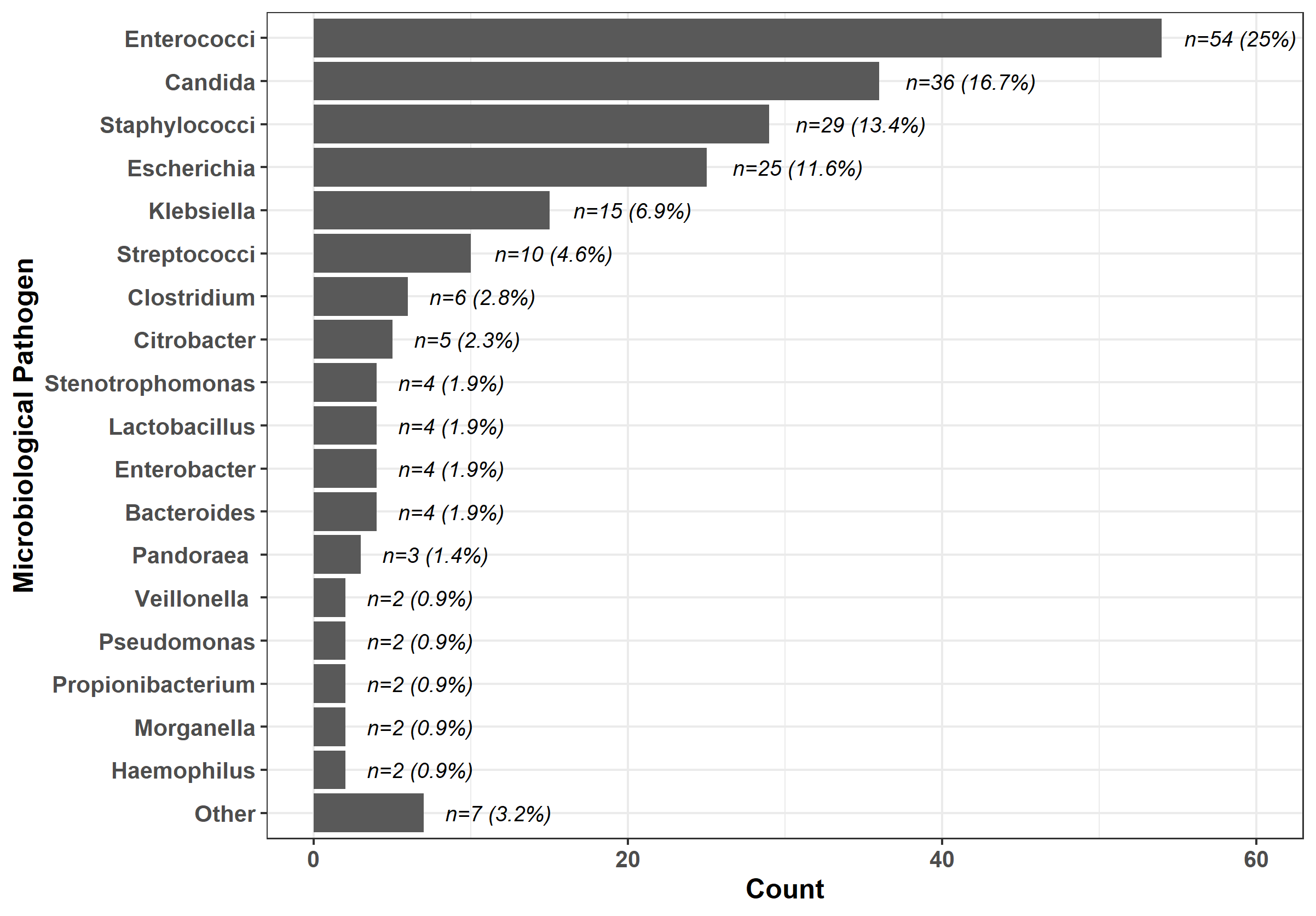

Supplement: Supplementary file 1 [file diagnostics-11-00826-s001.zip › diagnostics-1199423-supplementary.tiff]
